# Cycling of people with a lower limb amputation in Thailand

**DOI:** 10.1371/journal.pone.0220649

**Published:** 2019-08-02

**Authors:** Jutamanee Poonsiri, Rienk Dekker, Pieter U. Dijkstra, Yasmin Nutchamlong, Chanapak Dismanopnarong, Chiraphan Puttipaisan, Samai Suakonburi, Pensupa Pimchan, Juha M. Hijmans, Jan H. B. Geertzen

**Affiliations:** 1 University of Groningen, University Medical Center Groningen, Department of Rehabilitation Medicine, Groningen, The Netherlands; 2 Sirindhorn School of Prosthetics and Orthotics, Faculty of Medicine Siriraj Hospital, Mahidol University, Bangkok, Thailand; 3 University of Groningen, University Medical Center Groningen, Department of Oral and Maxillofacial Surgery, Groningen, The Netherlands; 4 Prosthetic and Orthotic Unit, Orthopaedic Department, Veterans General Hospital, Bangkok, Thailand; 5 Rehabilitation Medicine Department, Prosthetic and Orthotic Unit, King Chulalongkorn Memorial Hospital, Bangkok, Thailand; 6 Rehabilitation Department, Lerdsin Hospital, Bangkok, Thailand; 7 Prosthetic and Orthotic Unit, Orthopaedic Department, Phramongkutklao Hospital, Bangkok, Thailand; Holland Bloorview Kids Rehabilitation Hospital, CANADA

## Abstract

**Aim:**

To investigate cycling participation and barriers, and facilitators in adults with a lower limb amputation in Thailand.

**Method:**

Questionnaires were given to 424 adults with uni/bilateral lower limb amputation from midfoot to hip disarticulation level at five public hospitals in Bangkok and prosthetic mobile units in Thailand. Participant characteristics were summarized using descriptive statistics. Variables associated with cycling (p<0.1) were entered in a logistic regression model.

**Results:**

Participants who cycled (46.7%, N = 197), mostly used their walking prostheses (91.9%, n = 188). Of cyclists, 92.4% had cycled before the amputation. Cyclists started cycling after the amputation by themselves (86.7%) mostly in order to increase/maintain health (67.0%). Most cyclists cycled on quiet roads. The most frequent destination was shops/market (64.1%). More facilitators were reported than barriers. Most reported barriers were related to health problems and negative attitudes toward cycling. Most reported facilitators were related to perceived health benefits and positive attitude toward cycling. The likelihood of cycling after the amputation increased in people who cycled before the amputation, were amputated lower than the knee, used a prosthetic foot with axis/axes, were amputated due to trauma, had income higher than 415 euro/month, and who reported a higher numbers of facilitators.

**Conclusion:**

After a lower limb amputation, nearly half of people cycled. People with a below knee amputation due to trauma with prior cycling experience and higher income tended to cycle after the amputation. People who perceived more facilitators were more likely to cycle. Although cyclists could use a walking prosthesis to cycle, a prosthetic foot with a greater range of motion than the SACH increased the cycling likelihood.

## Introduction

Cycling is one of the common activities of people with a lower limb amputation (LLA), as a recreational activity, sport, physical activity and way of transportation [1–6]. Cycling is joint friendly [7,8], and is a moderate or vigorous intensity aerobic physical activity promoting health [9]. Dutch non-disabled commuters perceived time and distance between home and work as major barriers to cycle, while perceived health benefits positively influenced participation in cycling [10]. Australians without a LLA reported that inadequate continuous cycle infrastructure and danger from motorists were major barriers for public cycling [11]. Studies in people with a LLA, focused mainly on factors related to prosthetic or bicycle components influencing cycling [12–15]. A prosthetic socket made of thigh corset created skin abrasions during cycling [12]. Adjusting the bicycle crank arm allowed people with knee flexion limitation to cycle [13] or to reduce cycling asymmetry [14]. A stiff prosthetic foot reduced the cycling asymmetry and increased cycling performance [15]. Older aged people with an amputation and people with transfemoral amputation cycle less [2,6]. Influence of natural environment and infra-structure on cycling participation has not been investigated in people with a LLA [16].

Cycling has been promoted in Thailand for health benefits [17], tourists’ promotion [18], sustainable non-motorized transportation [19], and national events [20,21]. In Thailand, over 20,000 people have a LLA [22], but there is scarce information about their cycling participation. Since lifestyles of people with a LLA in other countries cannot be generalized to Thai people, it is not known whether cycling is a favored activity in Thai people with a LLA. The primary aim of this survey was therefore to analyze the cycling participation, frequency, duration, and reason of cycling in people with a LLA in Thailand. The secondary aim was to investigate cycling barriers, and facilitators in people with a LLA.

## Materials and methods

### Participants

Inclusion criteria for the survey were age older than 18 years, having had unilateral or bilateral LLA for at least 6 months in which the level of amputation ranged from midfoot to hemipelvectomy, able to read, write and speak Thai and having given written informed consent.

For sample size calculation, we estimated an expected percentage of cyclists of 50% which we wanted to estimate with an error margin of 5% resulting in a minimum sample size of 385 [23]. To compensate for missing data, an extra 10% was added to the target sample size resulting in a targeted sample of 424.

Participants were recruited via posters and direct invitation from Sirindhorn School of Prosthetics and Orthotics at Siriraj Hospital, Veterans General Hospital, Lerdsin Hospital, King Chulalongkorn Memorial Hospital and Phramongkutklao Hospital in Bangkok from February to July 2018. Additionally, participants who visited mobile units of the Veterans General Hospital and the Sirindhorn National Rehabilitation Institute, Ministry of Public Health were recruited in the same way. Mobile units travel to locations outside Bangkok as a service given to people with a LLA.

### Measures

A questionnaire was used to determine cycling participation and barriers and facilitators for cycling (S1 Appendix). The questionnaire consisted of six parts. Part 1 assesses the daily prosthesis, shoes and walking aids, filled out by the prosthetist. The other parts were filled out by the participants. Part 2 and Part 3 were modified from the questionnaires used for sports participation of people with LLA [24] and visual impairment [25]. These parts asked for basic participant characteristics such as gender, age, date of birth, cause of amputation, income, and education. Part 4, developed for this study, included questions about reasons for cycling, frequency, duration, intensity, and location of cycling, and use of cycling devices and /or bicycles. Part 5 includes questions about cycling barriers and facilitators which are based on previous studies [25–27]. Six subscales of Barriers to Physical Activity Questionnaire for People with Mobility Impairments (BPAQ-MI) were used: (1) health, (2) beliefs and attitudes, (3) family, (4) friends, (5) community built environment, and (6) safety [26]. Two subscales not relevant for this study, (fitness center built environment and staff/program/policy) were not used. The internal consistency of BPAQ-MI is good [26]. Knowledge and cost relating items from Physical Activity and Disability Survey (B-PADs) [27] were added. Items related to cycling barriers for transportation and leisure were used from a reliable instrument included (1) lack of safety, (2) poor quality of streets, (3) lack of dressing rooms (changing clothes/having a shower), (4) lack of a safe parking space for the bicycle, (5) intense traffic, (6) too much pollution, (7) lack of willingness, (8) unfavorable climate (sun, rain, cold), (9) not owning a bicycle, (10) distance to destinations, and (11) fear of accidents [28]. Finally, items related to cycling as an occupation such as work and competition were also added [25]. Questions [25–28] were adapted and wording was modified to match cycling.

All questions were translated from English to Thai by the first author [JP] and backward translated into English by another author who is fluent in Thai and English [YN] and was blinded for the Original English version [29]. The back translation was compared with the original English version. Discrepancies were reviewed and adjustments in the Thai questionnaire were made [29]. After the questionnaire had been filled out, it could be returned in a sealed envelope on the same day or sent the stamped- on envelope back by post within 2 weeks.

The ethical committees and/or hospitals approved this research. The project codes approved by the ethical committees of Siriraj Hospital, Lerdsin Hospital, King Chulalongkorn Memorial Hospital and Phramongkutklao Hospital are SIRB819/2560(EC4), 611010, IRB628/60, and IRBRTA133/2561 respectively.

### Data analysis

Similar barriers and facilitators were grouped according to BPAQ-MI [26]. Other items were grouped into subgroups; weather, pollution, destination, knowledge, cost, equipment, work, winning/competition, and other. As a result, 42 potential barriers were grouped into 12 barriers sub-groups. Additionally, 42 potentials facilitators were grouped into 14 sub-groups. If a participant selected several items from the same subgroup of facilitators or barriers, it was counted as one in the data analysis. Descriptive statistics were used to summarize the participants’ characteristics and participation level. Categorical variables were described as numbers and percentages. Continuous variables were described as mean and standard deviation (SD) or median and interquartile range as appropriate. Factors associated with bicycling participation were univariately explored. Factors significantly associated with bicycling participation (p<0.1) were entered into the logistic regression model. Step wise logistic regression was used, if a variable increased the model fit significantly or if the regression coefficient was significant, the variable was included in the model.

## Results

### Participants characteristics

In total 424 persons with a LLA participated. Two persons were removed from data set, one because of not having an amputation, but a proximal femoral focal deficiency, the other because of age less than 18 years resulting in a total of 422 participants. Most of participants were male (78.7%) with the mean age of 54.7± 13.1 years (Table 1). Participants came from 49 of the 77 Thai provinces. The majority of participants were recruited from Veterans General Hospital and the mobile unit (45.5%).

**Table 1:** Participant characteristics (n = 422).

**Characteristic (valid observations)**		**Mean**	**SD**
Age (years) (399)		54.7	13.1
Body weight (kg) (400)		65.5	11.3
Height (cm) (399)		165.8	7.7
Body Mass Index (kg/m^2^) (399)		23.8	3.7
Months since amputation (127)		258.6	185.8
		**n**	**%**
Gender (422):	Female	90	21.3%
Living area (422)			
Bangkok metropolitan		165	39.1%
Living circumstance (367)			
Single		39	10.6%
Parents		37	10.1%
Couple with children		189	51.5%
Couple with no children		66	18.0%
Relatives/ others		36	9.8%
Education (419)			
No		10	2.4%
Basic		177	42.2%
Did not complete high school		61	14.6%
High school		90	21.5%
College		36	8.6%
Bachelor		41	9.8%
Master		4	1.0%
Employment (414)			
Employed		105	25.4%
Self-employed		151	36.5%
Out of work& looking		13	3.1%
Out of work but not looking		83	20.0%
Student		5	1.2%
Retired		48	11.6%
Unable to work		9	2.2%
Monthly income (USD)* (416)			
Under 470		157	37.7%
470–945		134	32.2%
945–1,575		67	16.1%
1,575–3,145		18	4.3%
Over 3,145		3	0.7%
Do not wish to answer		37	8.9%
Level in unilateral amputees (407)			
HD		2	0.5%
TF		102	25.1%
KD		6	1.5%
TT		284	69.8%
AD		8	2.0%
MF		5	1.2%
Bilateral Amputation** (422)		15	3.6%
Cause of amputation (422)			
Cardiovascular disease		15	3.6%
Diabetes		44	10.6%
Accident		306	73.6%
Cancer		19	4.6%
Congenital		7	1.7%
Other causes		25	6.0%
Walking aid (422)			
None		346	82.0%
Walker		7	1.7%
Point cane		27	6.4%
Axillary crutch		28	6. 6%
Elbow crutch		1	0.2%
Other gait aid		13	3.1%

Valid observations = number of participants answering the question, SD = Standard Deviation

*1 USD = 31.8 Baht. Income 470–945 USD was considered average. Income below and above 470–945 USD was considered low and high respectively. HD = hip disarticulation, TF = transfemoral, KD = knee disarticulation, TT = transtibial, AD = ankle disarticulation, MF = mid foot

**Level of LLA (left–right) in 15 bilateral people with amputation are 7(TT-TT), 3(TF-TF), 1(HD-HD), 1(TT- KD), 1(TT- AD), 1(TF–TT), 1(TT-MF)

### Cyclists’ characteristics and factors associated with cycling

In total 46.7% of participants cycled after LLA. Cyclists were more likely to be male than non-cyclists. Additionally, they were heavier and taller than non-cyclists. Cyclists were more frequently employed and had higher income than non-cyclists. Cyclists had more frequently a unilateral LLA due to trauma, had been amputated for a longer period of time, amputated at a more distal level, and used a lighter prosthesis than non-cyclists. Cycling experience prior to the LLA, prosthetic systems and prosthetic feet of cyclists also differ from non-cyclists (Table 2).

**Table 2:** Characteristics of cyclists and non-cyclists.

**Characteristic** **(cyclists / non-cyclists)**	**Cyclist** **(n = 197)**		**Non-cyclists** **(n = 225)**		**Test statistic**^#^	**p**	**95%CI**
	**Mean**	**SD**	**Mean**	**SD**			
Age (years) (187/212)	54.3	12.6	55.0	13.5	-0.6	0.567	-0.8(-3.3, 1.8)
Body weight (kg) (186/214)	67.2	10.5	64.1	11.8	2.8	0.005	3.2(1.0, 5.4)
Height (cm) (187/212)	166.7	7.5	165.0	7.8	2.2	0.025	1.7(0.2, 3.2)
BMI (kg/m^2^) (184/209)	24.2	3.5	23.5	3.8	2.0	0.041	0.8(0.0, 1.5)
Months after amputation (58/69)	321.8	153.9	205.5	194.4	3.8	<0.001	116.3(55.1, 177.5)
Prosthetic weight (kg) (114/122)	2.2	0.7	2.8	1.3	-4.2	<0.001	-0.6(-0.8, -0.3)
		**n**	**%**	**n**	**%**	**Test statistic**^**#**^	**p**	
Gender (197/225): Female	32	16.2	58	25.8	5.7	0.017	
Living area (197/225): Bangkok metropolitan	72	36.5	93	41.3	1.0	0.315	
Living circumstance (181/186)							
Single	17	9.4	22	11.8	2.6	0.613	
Parents	15	8.3	22	11.8			
Couple with children	100	55.2	89	47.8			
Couple with no children	33	18.2	33	17.7			
Relatives/others	16	8.8	20	10.8			
Education (197/225):							
Under highschool	85	43.1	102	45.3	0.2	0.652	
Highschool to higher education	112	56.9	123	54.7			
Employment status (194/228):							
Unemployed	34	17.5	71	32.3	12.0	0.002	
Employed	134	69.1	122	55.5			
Student/retired	26	13.4	27	12.3			
Monthly income* (USD) (195/221):							
Under 470	45	23.1	112	50.7	34.4	<0.001	
470–945	78	40.0	56	25.3			
>945	53	27.2	35	15.8			
Do not wish to answer	19	9.7	18	8.1			
Have underlying disease (197/225)	96	48.7	125	55.6	2.0	0.161	
Level of amputation (197/225):							
KD,TF,HD	30	15.2	86	38.2	29.3	<0.001	
TT, AD, MF	167	84.8	139	61.8			
Uni/bilat (197/225): Unilateral	194	98.5	213	94.7	4.4	0.035	
Reason of amputation**(194/222):							
Cardiovascular	3	20.5	12	5.4	32.2	<0.001	
Diabetes	7	3.6	37	16.7			
Accident	166	85.6	140	63.1			
Cancer	6	3.1	13	5.9			
Congenital	3	1.5	4	1.8			
Other	9	4.6	16	7.2			
Cycling before amputation (197/225)	182	92.4	147	65.3	44.7	<0.001	
Use of gait aid (197/225)	71	36.0	92	40.9	1.0	0.307	
Experienced cycling barrier (197/225)	102	51.8	140	62.2	4.7	0.030	
Prosthetic system (188/199):							
Endoskeletal	69	36.7	97	48.7	5.7	0.017	
Exoskeletal	119	63.3	102	51.3			
Prosthetic foot (174/196):							
SACH	125	71.8	158	80.6	3.9	0.047	
Single axis/ Dynamic	49	28.2	38	19.4			
TT, AD, MF socket (155/124):							
PTB	114	72.2	81	65.3	2.08	0.568	
PTBSC	39	24.7	38	30.6			
PTBSCSP	2	1.3	3	2.4			
Other	0	0.0	2	1.6			
TT, AD, MF liner (162/125):							
None	13	8.0	6	4.8	8.9	0.026	
Pelite	142	87.7	108	86.4			
Silicone	7	4.3	5	4.0			
Silicone,pelite	0	3.4	6	4.8			
TT, AD, MF suspension (139/ 111):							
Cuff	117	84.2	83	82.2	1.2	0.95	
Sleeve	3	2.2	2	2.0			
Pin	1	0.7	1	1.0			
Silesian	3	2.2	4	4.0			
Other	15	10.8	11	10.9			
HD,TF,KD socket (27/ 74):							
Ischial containment	3	12.0	9	12.2	2.5	0.402	
Quadrilateral	22	88.0	64	86.5			
Other	2	7.4	1	1.4			
HD,TF,KD liner (19/ 65):							
None	15	78.9	60	92.3	4.4	0.092	
Pelite	3	15.8	5	7.7			
Silicone	1	5.3	0	0.0			
HD,TF,KD knee (24/70):							
None	2	7.4	5	6.8	3.3	0.522	
Weight activate	5	18.5	22	30.1			
Four bar	17	63.0	41	56.2			
Manual	0	0.0	2	2.7			
HD,TF,KD suspension (28/ 77):							
Cuff	1	3.6	2	2.6	6.0	0.133	
Pin	0	0	1	1.3			
Silesian	18	64.3	64	83.1			
Suction	7	25.0	8	10.4			
Other	2	7.1	2	2.6			
	**Median**	**(IQR)**	**Median**	**(IQR)**	**Test statistic**^**#**^	**p**	
Number of barriers (197/225)	0	(0;2)	1	(0;2)	20.3	0.120	
Number of facilitators (197/225)	4	(2;11)	1	(0;3)	31.7	<0.001	

# test statistic for means is t and for percentages it is χ^2^; CI = confidence interval; kg = kilogram; cm = centimeter; m = meter; The minimum expected count is 1.41; HD = hip disarticulation, TF = transfemoral, KD = knee disarticulation, TT = transtibial, AD = ankle disarticulation, MF = mid foot

*1 USD = 31.8 Baht. Income 470–945 USD was considered average. Income below and above 470–945 USD was considered low and high respectively.

** Reason of amputation for bilateral amputee was counted as one if it is the same for both left and right side. IQR = Interquartile Range; PTB = patellar tendon bearing; PTBSC = PTB and supra condylar; PTBSCSP = PTBSC and suprapatellar

### Reasons of cycling

In total 84.7% of the participants who cycled after the LLA did so because they wanted it themselves (Table 3). Main reasons for cycling were to increase/maintain health/physical fitness (67.0%), increase/maintain strength (59.9%), transport/ commute from place to another place (46.7%), have fun/ relaxation (31.5%), control weight (26.4%) and increase independence (25.4%). In total, 77 participants cycled for both recreation and transportation (Table 3). For recreational purpose, most people cycled alone (77.8%). None of cyclists cycled with fellow amputees, trainer/therapist, or others (Table 4).

**Table 3:** Motivators and reasons of cycling.

	n	%
**Motivators:**		
** **I want to ride the bicycle myself	166	84.7
** **Friends	20	10.3
** **Family/partner/children	15	7.7
** **Doctor/Rehabilitation practitioner	8	4.1
** **Physiotherapist	5	2.6
** **Caretaker	3	1.5
** **Fellow amputees	1	0.5
** **Occupational therapist	1	0.5
** **Internet	1	0.5
** **Television	1	0.5
** **Other	1	0.5
** **Prosthetist made amputees cycle	0	0.0
**Cycling reasons:**		
** **Increase/ maintain health/physical fitness	132	67.0
** **Increase/maintain strength	118	59.9
** **Transport/ commute from one place to another place	92	46.7
** **Have fun/ relaxation	62	31.5
** **Control weight	52	26.4
** **Increase independence	50	25.4
** **Increase/maintain social contacts	37	18.8
** **Accept disability	33	16.8
** **Increase self- confidence	32	16.2
** **Learn new skills	24	12.2
** **Learn how to deal with disability/assistive device	19	9.6
** **Work (e.g. deliver some products)	13	6.6
** **Compete in the national level	2	1.0
** **Compete in the international level	2	1.0
** **Other reason	1	0.5

**Table 4:** Cycling intensity, frequency, path, and destination according to the purpose of cycle.

	**Fun/relaxation/exercise**		**Transportation**	
	**n = 134**		**n = 92**	
	**Median**	**(IQR)**	**Median**	**(IQR)**
Frequency (times a day)	0.8	(0.1;2.0)	1.0	(0.3;2.0)
Duration (minute per ride)	30.0	(15.0;30.0)	20.0	(12.5;30.0)
Distance (kilometers per ride)	3.0	(1.0;5.0)	2.0	(1.0;4.0)
	**n**	**%**	**n**	**%**
Intensity: Moderate	123	91.1	4	4.3
Vigorous	11	8.1	76	82.6
With: Alone	105	77.8	NA	NA
** **Family	23	17.0	NA	NA
** **Friends	28	20.7	NA	NA
** **Club/association member	7	5.2	NA	NA
On: Quiet roads(no bike lanes)	66	48.9	50	54.3
** **On-road bicycle lanes	24	17.8	17	18.5
** **Shared paths (pedestrians and bicycles)	19	14.1	11	12.0
** **Offroad bicycle path	11	8.1	10	10.9
** **Foot paths	16	11.9	14	15.2
** **Busy roads (no bike lanes)	15	11.1	14	15.2
** **Fitness	3	2.2	NA	NA
** **Park	24	17.8	NA	NA
** **Rehabilitation center/ hospital	2	1.5	NA	NA
** **Other	10	7.4	5	5.4
To: Shops/ market	NA	NA	59	64.1
** **Visit friends/family	NA	NA	45	48.9
** **School/university/ work	NA	NA	6	6.5
** **Train/ bus/ boat station	NA	NA	3	3.3
** **Temple/church	NA	NA	15	16.3
** **Other	NA	NA	16	17.4

NA = not applicable.

### Devices and bike used for cycling

Among the people who cycled, a daily walking prosthesis was used most often (91.9%). Cyclists cycled with daily walking shoes (97.2%). The top three commonly used bicycles were grandma bike (44.9%), mountain bike (17.3%), and BMX (14.1%) (Table 5).

**Table 5:** Daily prosthesis, walking aids and shoes of cyclists (n = 188).

		n	%
The use of prosthesis:	Not use prosthesis	6	3.2
	Use prosthesis	178	95.7
	Use and not use prosthesis	2	1.1
Types of prosthesis used:	Adapted prothesis	7	3.8
	Daily prosthesis	170	91.9
	Not use prosthesis	6	3.2
	Other	2	1.1
Type of bicycle used:	Adapted bicycle	8	4.3
	Grandma bike	83	44.9
	Mountain bike	32	17.3
	BMX	26	14.1
	Stationary bike	8	4.3
	Touring bike	4	2.2
	3 wheel	3	1.6
	Other	1	0.5
	Used more than 1 types of bicycles	20	10.8
Types of shoes used:	Adapted shoes	2	1.1
	Daily shoes	174	97.2
	Not wear shoes	2	1.1
	Cycling shoes	1	0.6

### Barriers and facilitators for cycling

Both cyclists and non-cyclists reported barriers and facilitators for them to start or maintain cycling (Table 6, S2 Appendix). In general, facilitators were more often reported than barriers. Cyclists reported more facilitators and barriers than non-cyclists and a larger number of reported facilitators associated with cycling participation.

**Table 6:** Cycling barriers and facilitators of cyclists and non-cyclists.

	Cyclists (n = 197)		Non-cyclists (n = 225)		Chi-square test	p
	n	%	n	%		
**Barriers:**						
Health: no energy, pain, wound, discomfort, poor health condition	51	25.9	49	21.8	1.0	0.322
Negative attitude toward cycling	42	21.3	67	29.8	3.9	0.048
Support/encouragement from friends, family, care taker, amputee buddies, medical staff	7	3.6	7	3.1	0.1	0.800
Built environment: dressing room, rest areas, potholes on street, bike parking space/ path	29	14.7	13	5.8	9.4	0.002
Safety (crime, speed and number of cars, traffic light, loose dogs)	29	14.7	13	5.8	9.4	0.002
Weather	12	6.1	2	0.9	8.9	0.003
Pollution	10	5.1	4	1.8	3.6	0.059
High cost for training/ bike/ prosthesis	8	4.1	7	3.1	0.3	0.599
Lack of cycling skill /knowledge how and where to cycle	4	2.0	26	11.6	14.4	<0.001
Equipment: Not owning a bike/ inappropriate prosthesis/bike	16	8.1	43	19.1	10.5	0.001
Distance to destination is too far / too close	10	5.1	7	3.1	1.0	0.306
Others	22	11.2	32	14.2		
**Facilitators:**						
Health: increase / maintain physical fitness, increase / maintain strength, control weight	134	68.0	91	40.4	32.1	<0.001
Attitude: fun/ relax, increase/maintain self-confidence, learn new skills, increase/maintain independence, accept disability, learn how to deal with disability/ assistive device, increase/maintain social contacts	115	58.4	60	26.7	43.5	<0.001
Support/encouragement from family, friends, personal care taker, medical/rehabilitation practitioners, buddies with amputation	27	13.7	26	11.6	0.4	0.506
Competition/ winning	3	1.5	5	2.2	0.3	0.729
Work	13	6.6	5	2.2	4.9	0.026
Built environment	48	24.4	29	12.9	9.3	0.002
Good weather	56	28.4	26	11.6	19.1	<0.001
No pollution	44	22.3	14	6.2	23.0	<0.001
Safety	50	25.4	29	12.9	10.8	0.001
Cost and availability of bike/ prosthesis	26	13.2	37	16.4	0.9	0.350
Knowing how and where to cycle	46	23.4	30	13.3	7.1	0.008
Satisfied with a current prosthesis/ bicycle	59	29.9	34	15.1	13.5	<0.001
Appropriate distance to destination	57	28.9	27	12.0	18.9	<0.001

For cyclists, the barriers were less often reported compared to the facilitators. The barriers that were reported more than 10% among cyclists were health (25.9%), attitude (21.3%), safety (14.7%), built environment (14.7%), and other reasons (11.2%). Top 10 reported facilitators were perceived health benefit (68.0%), positive attitude toward cycling (58.4%), satisfied with prosthesis and bicycle (29.9%), perceived appropriate distance (28.9%), perceived safety (25.4%), appropriate built environment (24.4%), having cycling knowledge or skill (23.4%), no pollution (22.3%), adequate support (13.7%), and affordable cost of equipment or training (13.2%).

For non-cyclists, barriers by rank were attitude (29.8%), health (21.8%), equipment (19.1%), other reasons (14.2%), and knowledge (11.6%). Facilitators for non-cyclists reported by rank were health (40.4%), attitude (26.7%), affordable cost of equipment or training (16.4%), satisfied with prosthesis and bicycle (15.1%), knowledge (13.3%), safety (12.9%), built environment (12.9%), distance (12.0%), and good weather (11.6%).

### Factors associated with cycling participation in a logistic regression

In the logistic regression, the effects of height, body weight, BMI, months since amputation, prosthetic weight, gender, employment status, income, reasons and levels of a LLA, history of cycling before a LLA and number of facilitators on the likelihood that participants ride the bike were analyzed. The model explained 38.9% (Nagelkerke R^2^) of the variance in riding the bike and correctly classified 76.5% of cases. The chance of people cycling after the amputation was 9.2 times higher for those who cycled before the LLA compared to those who did not. People with below knee amputation were 4.5 times more likely to cycle than knee disarticulation and above knee amputation. Participants who used a single axis or dynamic foot cycled 1.9 times more likely to cycle than those using a SACH foot. A larger number of the reported facilitators and a higher income were associated with an increased likelihood of cycling (Table 7).

**Table 7:** Logistic regression of variables associated with cycling participation.

	B	S.E.	Sig.	Exp(B)	95% C.I.for EXP(B)	
					Lower	Upper
Below knee amputation^a^	1.500	0.328	<0.001	4.480	2.355	8.525
Traumatic amputation^b^	0.947	0.345	0.006	2.579	1.311	5.074
Foot with axis/s^c^	0.657	0.326	0.044	1.929	1.019	3.651
Total number of facilitators^d^	0.084	0.021	<0.001	1.087	1.044	1.133
Cycle before amputation^e^	2.221	0.399	<0.001	9.214	4.214	20.145
Higher income^f^	0.631	0.278	0.023	1.880	1.090	3.242
Constant	-5.417	0.704	<0.001	0.004		

a: above knee amputation is the reference group

b: non-traumatic amputation is the reference group

c: SACH foot is the reference group

d: not reported any facilitator is the reference group

e: not-cycling before is the reference group

f: income lower than 470 USD is the reference group, Exp(B): odds ratio

## Discussion

### Cycling participation after LLA

This study evaluated cycling participation of Thai adults with a LLA. Most cyclists with a LLA have cycled before a LLA. About 45% of people who ever cycled, stopped cycling after a LLA. After a LLA, people cycled for different reasons. The top cycling purposes were recreation, in order to increase or maintain physical fitness.

Perceived poor health condition and negative attitude were the top two most reported barriers for all participants to cycle. The third most often reported barrier among cyclists was inappropriate/ inadequate built environment and safety, while for non-cyclists, the third most often reported barrier was related to equipment- not owning the bicycle/ inappropriate bicycle/prosthesis. Most barriers were indeed reported by cyclists rather than non-cyclists (except attitude, knowledge, equipment and other relating barriers). Our finding is similar to other studies that after a LLA, people stopped cycling [6], reduced sport participation, or changed life-style [1–5]. Some participants described that they have bilateral amputation, have suffered from a stroke, hip, knee or balance problems making it impossible to cycle. Common destinations were shops or friend’s place. For both transportation and recreation, the most popular place to cycle on was a quiet road. This outcome is probably why the living location (Bangkok city/ urban/rural) is not related to participation, as the people with a LLA tend to cycle for a short distances and not on the busy road with cars, motorcycles, or other vehicles. Perceived lack of or inappropriate built environment, negative attitude, and safety could contributed to a short distance of cycling and cycling on the quiet road. To stimulate cycling participation the government should improve built-environment and safety to increase the duration and distance of cycling by means of separating cycling lanes from cars or motorcycles, installing traffic lights for bicycles, strictly limit car speed in shared road, or control of loose dogs.

The majority of cyclists used a daily prosthesis (exoskeleton system, PTB with Pelite liner or QL (quadrilateral) socket design, SACH foot with the cuff or Silesian belt (suspensions) for cycling. Cyclists mostly used a grandma bike and regular shoes. Satisfaction with the prosthesis was more often reported as a facilitator in cyclists than in non-cyclists. Eight people did not use a prosthesis, and they thought they could not cycle. Prosthetic problems were reported as barriers including skin abrasion, foot slipping off the pedal, tighten of cuff suspension and problems of prosthetic knee flexion. We suggest that custom adjustments or change of prosthetic components may be needed to stimulate cycling participation.

### Cycling prior to LLA

Logistic regression shows that the factors associated with cycling after the LLA are; cycling prior to LLA, level of LLA, prosthetic foot, amputation cause, income, and total number of facilitators. Cycling experience prior to the LLA is the strongest predictor in cycling participation after the LLA. Previous research has also shown that participating in sports/ having a history of frequent vigorous physical activity [3] prior to the LLA increased the likelihood of sport participation after the LLA [24]. Probably having cycling skills prior to prior to LLA helps to cycle after the LLA. Additionally, knowledge related barrier and facilitator were significantly associated to cycling. These results are similar to those reported by studies in people after acquiring another disease or disability [3,27,30]. To promote cycling after a LLA, the rehabilitation team should inform people without prior cycling experience about positive effects of cycling and help them reduce barriers to allowing cycling.

### Level of LLA

People with TT, AD, and MF level are 4.5 times more likely to cycle than people with a more proximal level of amputation. This is in line with some studies showing that people with a TT amputation were more active and independent than people with a TF amputation [6,31]. One study reported that people with a TF amputation cycled more than a TT amputation [3]. The authors of that paper suggested that TF were more active possibly due to a more active profile prior to the LLA, higher education, socioeconomic level and more spare time after the LLA [3]. People with more proximal levels of amputation KD, TF, and HD also have to use prosthetic knee components. In our study there was no association between the prosthetic knee joint and cycling participation, although limited knee flexion ability was mentioned as a barrier. Greater energy expenditure is required to ambulate in people with a more proximal level of amputation than a distal level [32] and this may be the case in cycling too. However, it has not been shown that people with a more proximal level require more energy and effort to cycle.

### Prosthetic foot

When cycling, the required ankle range of motion is about 50° with a maximum of 13° dorsiflexion at the 90° crank position and 37° maximum plantar flexion at 285° crank position in healthy people [33]. However, this range cannot be achieved by SACH feet [34]. A greater ankle range of motion is possible with a single axis foot [35] or dynamic foot [34]. Participants also reported the prosthetic foot slipping off the pedal as a barrier. As a result, a foot/ankle with more ROM could increase in the likelihood of cycling participation. The SACH foot has been suggested to be a suitable foot for recreational cyclists [15]. However, the suggestion was based on a high intensity cycling test. Future studies are required to determine whether the SACH foot is indeed adequate for the recreational cycling.

### Amputation cause

A majority of participants were amputated due to accident (73.6%), and in cyclists this was 85.6%. This is in line with studies showing that people with a LLA resulting from trauma were more active than people with a LLA from peripheral vascular diseases [36]. Non-cyclists reported health-related barriers (lack of energy, pain, wound, discomfort while cycling and poor health conditions) as the second most reported barriers for them to cycle. About 45% of participants were from the Veterans General Hospital treating patients who were injured during active military service. Veterans remained physically active even after the LLA and usually got a LLA when they were young and physically fit [3]. Hence, a trauma-related cause of LLA may be a predictor of cycling because of a healthier profile of people with a LLA than disease related cause.

### Income

Income was a cycling predictor as people with a monthly income higher than 15,000 baht (approximately 470 USD) were more likely to cycle than people with lower incomes. The majority of cyclists used an ordinary bike with a daily walking prosthesis. For people who already have a bicycle, there are no extra costs involved to cycle. The results showed that more than 10% of cyclists have more than one bike. Cost related barriers and facilitators were also not significantly associated to cycling. It might be that people with higher income tend to have more active lifestyle and cycle more as suggested previously [3].

### Total number of facilitators

People who reported more facilitators were more likely to cycle. Although the number of facilitators reported by cyclists was greater than non-cyclists, several non-cyclists also reported facilitators in many topics particularly toward the health benefits of cycling. In spite of the frequently reported perceived health benefits, barriers such as negative attitude toward cycling may outweigh the benefits in non-cyclists. The item score showed that nearly 15% of non-cyclists reported being afraid of getting injured. Additionally, about 10% reported lack of motivation, feeling embarrassed to cycle and lack of reasons, but these items were infrequently reported among cyclists. It is likely that these personal factors limited people with a LLA to start or maintain cycling even though the perceived health benefits.

Health-related barrier was another frequently reported barrier. Cyclists often reported pain (12.2%) followed by discomfort while cycling (9.1%). These barriers could contribute to the distance or frequency of cycling. Surprisingly, 13.8% of non-cyclists also reported discomfort while cycling, so some non-cyclists have tried to cycle after a LLA. Pain and discomfort could be the result of using an inappropriate prosthesis or bicycle. However, these equipment factors were infrequently barriers but rather as facilitators for cyclists. Hence, it is possible that cyclists may cycle longer or more frequently if they have the appropriate equipment.

## Limitations

The majority of participants were male and with a transtibial amputation. In 2012, there were 24,798 people with a LLA in Thailand (73% was male), but there was information about level and causes of a LLA [22]. About 45 percent of the participants were veterans. The number of participants who refused to answer the questionnaire was not recorded. Therefore, generalization all people with a LLA in Thailand may be limited. Data from this study was self-reported and cycling participation (frequency, duration, and distance) may be over or under-reported. Some participants skipped some questions but all available data was analyzed including the incomplete questionnaires. All participants filled-in the questionnaire at the clinic or mobile unit and prosthetists were available for answering questions. This availability may have influenced answering tendencies. Participants reported more facilitators than barriers which could be a result of people who did not cycle did not realizing any barrier or experienced them and skipped the question “Have you experienced any barrier when cycling?” Because items underneath the barriers and facilitators are mirrored, still insight about factors associated to cycling is given in this study.

## Conclusions

Having cycled before a LLA is the important cycling predictor after a LLA. People with a LLA level lower than the knee joint, caused by trauma and / or with higher income are likely to cycle more than the others. People who perceived more facilitators were more likely to maintain or start cycling. Prosthetists should select a prosthetic foot with more range of motion for cyclists. Cycling training can be included in the rehabilitation program to increase cycling skill/knowledge and confidence, especially to the people who never cycled before a LLA. Adjusting the bicycle or prosthesis to match with the individual’s conditions may increase cycling duration and frequency.

## Supporting information

S1 Appendix Questionnaire. (DOCX)

S2 Appendix Item score of barriers and facilitators. (DOCX)
