# Supplementary material for: Cycling of people with a lower limb amputation in Thailand
Source: PLoS One. 2019 Aug 2;14(8):e0220649. doi: 10.1371/journal.pone.0220649 (PMC6677311; doi:10.1371/journal.pone.0220649)
Supplement: S1 Appendix — (DOCX) [file pone.0220649.s001.docx]

# S1 Appendix. Questionnaire.

| **Part 1: Daily prosthesis and shoe** (**prosthetist will fill out)  In this part, the prosthetist will assess the daily prosthesis, shoes, and walking aids that the patient uses for walking by ticking(✓) in the box or writing down the information in the blank space(____). Thank you very much for your help! ☺ | | | | | |
| --- | --- | --- | --- | --- | --- |
|  | - Left | | | - Right | |
| Amputation level | - Hemipelvectomy - Hip disarticulation - Trans-femoral - Knee disarticulation - Trans-tibial - Ankle disarticulation - Midfoot amputation - Other,   namely:_____________ | | | | - Hemipelvectomy - Hip disarticulation - Trans-femoral - Knee disarticulation - Trans-tibial - Ankle disarticulation - Midfoot amputation - Other,   namely:_____________ |
| Prosthesis System | - Endoskeletal - Exoskeletal | | | | - Endoskeletal - Exoskeletal |
| Socket | € PTB (patellar tendon bearing)  € PTB- SC (supracondylar)  € PTB- SCSP (supracondylar suprapatellar)  € ICS (Ischial Containment Socket)  € QL Quadrilateral socket     - Other,   namely:_________________ | | | | € PTB  € PTBSC  € PTBSCSP  € ICS    € QL   - Other, namely:_________________ |
| Liner | € None   - Pelite/foam   € Silicone   - Other,   namely:_________________ | | | | € None   - Pelite/foam   € Silicone   - Other, namely:_________________ |
| Suspension | € Cuff  € Sleeve  € Pin shuttle lock  € Silesian belt  € Suction   - Other, namely:___________________________________________ |  | | | € Cuff  € Sleeve  € Pin shuttle lock  € Silesian belt  € Suction  Other, namely:___________________________________________ |
| Hip joint | \| € None   - Other,   namely:_________________ \| € None   - Pelite/foam   € Silicone   - Other, namely:_________________ \| \| --- \| --- \| | | \| € None   - Other,   namely:_________________ \| € None   - Pelite/foam   € Silicone   - Other, namely:_________________ \| \| --- \| --- \| | | |
|  | - Left | | | - Right | |
| Knee joint | - None - Microprocessor controlled - Single axis weight activate - Four bar linkage - Manual lock    Other,  namely: __________________ |  | | | - None - Microprocessor controlled - Single axis weight activate - Four bar linkage - Manual lock    Other,  namely: __________________ |
| Foot | - SACH   € Single axis  € ESAR(Energy storage and return)   Other,  namely: __________________ | | | | - SACH   € Single axis  € ESAR   Other,  namely: __________________ |
| Number of stockinette/socks | - None - 1 layer - 2 layers - 3 layers - ____________layers | | | | - None - 1 layer - 2 layers - 3 layers - ____________layers |
| Weight of prosthesis | ________________________kg | | | | _____________________kg |
| Gait aid | - None - Walker  \| € single point cane   - axillary crutch - elbow crutch - Other,   namely:__________________ \| € single point cane   - axillary crutch - elbow crutch - Other, namely:__________________ \|  \| \| --- \| --- \| --- \| | | | | |
| Shoes | \| \| € \| 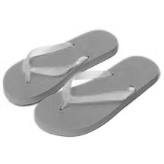 \| € \| 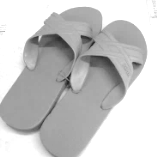 \| € \| 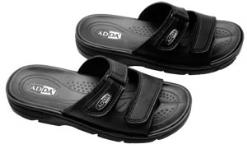 \| € \| 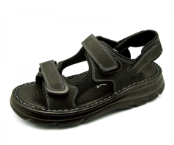 \| € \| 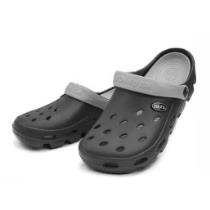 \| \| --- \| --- \| --- \| --- \| --- \| --- \| --- \| --- \| --- \| --- \| \| Y strap \| \|  \| X strap \| \| Adjustable dorsal strap \| Adjustable dorsal and heel straps \| \| Plastic clog \| \| \| € \| 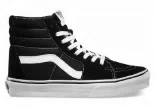 \| € \| 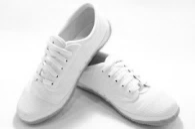 \| € \| 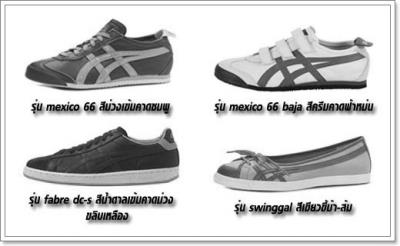 \| € \| 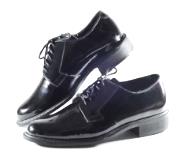 \| € \| 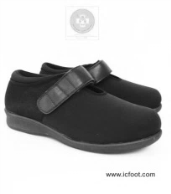 \| \| High top \| \| Below ankle \| \| Slip on \| \| Oxford \| \| Pre-Fabribcated  Diabetic shoe \| \|  - Other, namely:_________________________________ \| \| --- \| --- \| --- \| --- \| --- \| --- \| --- \| --- \| --- \| --- \| --- \| --- \| --- \| --- \| --- \| --- \| --- \| --- \| --- \| --- \| --- \| --- \| --- \| --- \| --- \| --- \| --- \| --- \| --- \| --- \| --- \| --- \| --- \| --- \| --- \| --- \| --- \| --- \| --- \| --- \| --- \|   Heel Heights: ________________________cm | | | | |

From this page until the end, the questionnaire will be filled in by people with lower limb amputation. Please tick (✓) in the box (€)of the response that most closely reflects your opinion or write down the answer in the blank space (____________).

Date of filling in the questionnaire: _____/ ______/______

**Part 2: General information**.

1. Date of birth : ______/ ______/ ______ (Age was asked instead of date at Siriraj Hospital )

2. Weight :____________Kilogram , Height: ____________Centimeter

3. Gender

| - male | - female |
| --- | --- |

4. Where do you live?

District:____________________ Province:__________________________

5. Whom do you live with?

- Single person household
- Living with parent/s
- Couple with children at home
- Couple, with no children at home
- Group household
- Other, namely:________________________________________

6. What is the highest level of education you have completed?

- No education
- Basic education
- Did not complete High School
- High School
- Some College
- Bachelor's Degree
- Master's Degree
- Advanced Graduate work or Ph.D.
- Other, namely:___________________________________________

**7. What is your employment status?**

- Employed
- Self-employed
- Out of work and looking for work
- Out of work but not currently looking for work
- Student
- Retired
- Unable to work because:____________________________________

8. Which of the following range your household monthly income falls?

- Under 15,000 Baht
- 15,000 – under 30,000 Baht
- 30,000 – under 50,000 Baht
- 50,000 – under 100,000 Baht
- Over 100,000 Baht
- Do not wish to answer

**Part 3: Questions regarding the amputation**

| 9. Do you have one or more of the following diseases (see question 10)?   - No. Go to question 11. - Yes  1. Which disease(s) do you have?  - Rheumatism - Cardio-vascular disease - Erosion of the joints - Bronchitis - Kidney disease - Other, namely: ________________________________________________ _____________________________________________________________   11. When did you have the last amputation? | | |
| --- | --- | --- |
| Left | Right |  |
| ______/ ______/ ______ | ______/ ______/ ______ |  |

12. What was the reason for the amputation?

| Left | Right |
| --- | --- |
| - Cardio-vascular disease - Diabetes - Accident - Cancer - Congenital/ from birth - Other: ___________________ | - Cardio-vascular disease - Diabetes - Accident - Cancer - Congenital/ from birth - Other: ___________________ |

**Part 4: Questions regarding cycling**

13. Did you cycle before your amputation?

| - No | - - Yes |
| --- | --- |

14. Do you cycle?

| - No. Go to 37 | - Yes |  |
| --- | --- | --- |
| 15. Who/what made you ride the bicycle after the amputation? (multiple answers are possible )   - Doctor /rehabilitation practitioner - Physiotherapist - Prosthetist - Occupational therapist - Family /partner/children - Friends - Caretaker - Fellow amputees - I want to ride the bicycle myself - Internet - TV - Other, namely:_____________________________ | | |

16. Why do you ride a bicycle? (Multiple answers are possible)

| I ride a bicycle  because I want to: | - Increase / maintain health/physical fitness - Increase/ maintain strength - Control weight - Have fun/ relaxation - Increase self-confidence - Learn new skills - Increase independence - Accept disability - Learn how to deal with disability/ assistive device - Increase/ maintain social contacts - Work (e.g. deliver some products) - Transport/commute from one place to another place - Compete in the national level - Compete in the international level |
| --- | --- |

17. Could you think of other reasons for you to cycle?

- No
- Yes: _________________________________________

| If you cycle for **fun/ relaxation/exercise** during the **last 6 months,** please answer question 18 to 23. |
| --- |
| 18. How often did you cycle:   - __________times /day - __________times /week - __________times / month - Less than once / month |
| 19. What was the mean duration?  ____________minutes/ time |
| 20. What was the mean distance?  ____________kilometers/time |
| 21. What was the cycling intensity?   - **Moderate:** take moderate physical effort and make you breathe somewhat harder than normal. - **Vigorous:** take hard physical effort and make you breathe much harder than normal. |
| 22. Whom did you cycle with?(multiple answers are possible) :   - Alone - Family - Friends - Fellow amputee persons - Trainer/ therapist - Club/association member - Other, namely:_____________________________ |
| 23. Where did you cycle at? (multiple answers are possible) :   - Quiet roads(no bike lanes) - On-road bicycle lanes - Shared paths (pedestrians and bicycles) - Off-road bicycle path - Foot paths - Busy roads(no bike lanes) - Fitness - Park - Rehabilitation center/ hospital - Other, namely:_____________________________ |

| If you **transport by cycling** during the **last 6 months,** please answer question 24 to 29. |
| --- |
| 24. How often did you cycle:   - __________times /day - __________times /week - __________times / month - Less than once / month |
| 25. What was the mean duration?  ____________minutes/ time |
| 26. What was the mean distance?  ____________kilometers/time |
| 27. What was the cycling intensity?   - **Moderate:** take moderate physical effort and make you breathe somewhat harder than normal. - **Vigorous:** take hard physical effort and make you breathe much harder than normal. |
| 28. Where did you cycle to?(multiple answers are possible) :   - Shops/ market - Visit friends/family - School/university/ work - Train/ bus/ boat station - Temple/church - Other, namely:_____________________________ |
| 29. Where did you cycle on? (multiple answers are possible) :   - Quiet roads(no bike lanes) - On-road bicycle lanes - Shared paths (pedestrians and bicycles) - Off-road bicycle path - Foot paths - Busy roads(no bike lanes) - Other, namely:_____________________________ |

30. Do you have a cycling prosthesis?

| - No. Go to 33 | - Yes |
| --- | --- |

31. When did you get this cycling prosthesis? ______/ ______/ ______

32. When cycling, do you use this cycling prosthesis?

| - No. Go to 33 | - Yes. Go to 34 |
| --- | --- |

33. When cycling , what kind of prosthesis do you use?

- An adapted daily prosthesis, describe how it is adapted: ______________________

__________________________________________________________________

- Use of daily prosthesis
- No use of prosthesis
- Other,namely:______________________________________________________________________________________________________________________________________________________________________________________________

34. When cycling , what kind of bicycle do you use?

- An adapted daily bicycle, please describe how it is adapted:

______________________________________________________________________________________________________________________________________

- Use of daily bicycle, tick the type of bicycle

| - 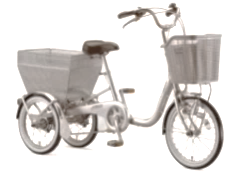   3 wheels   - 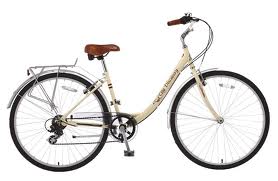Grandma’sbike | - 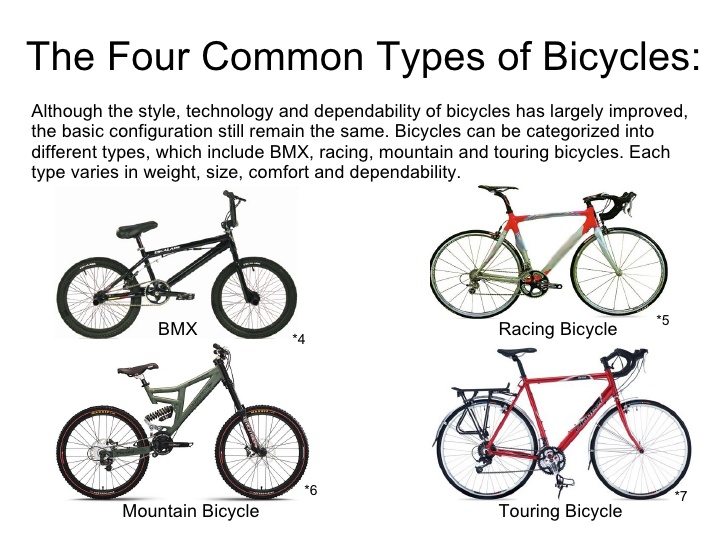 - 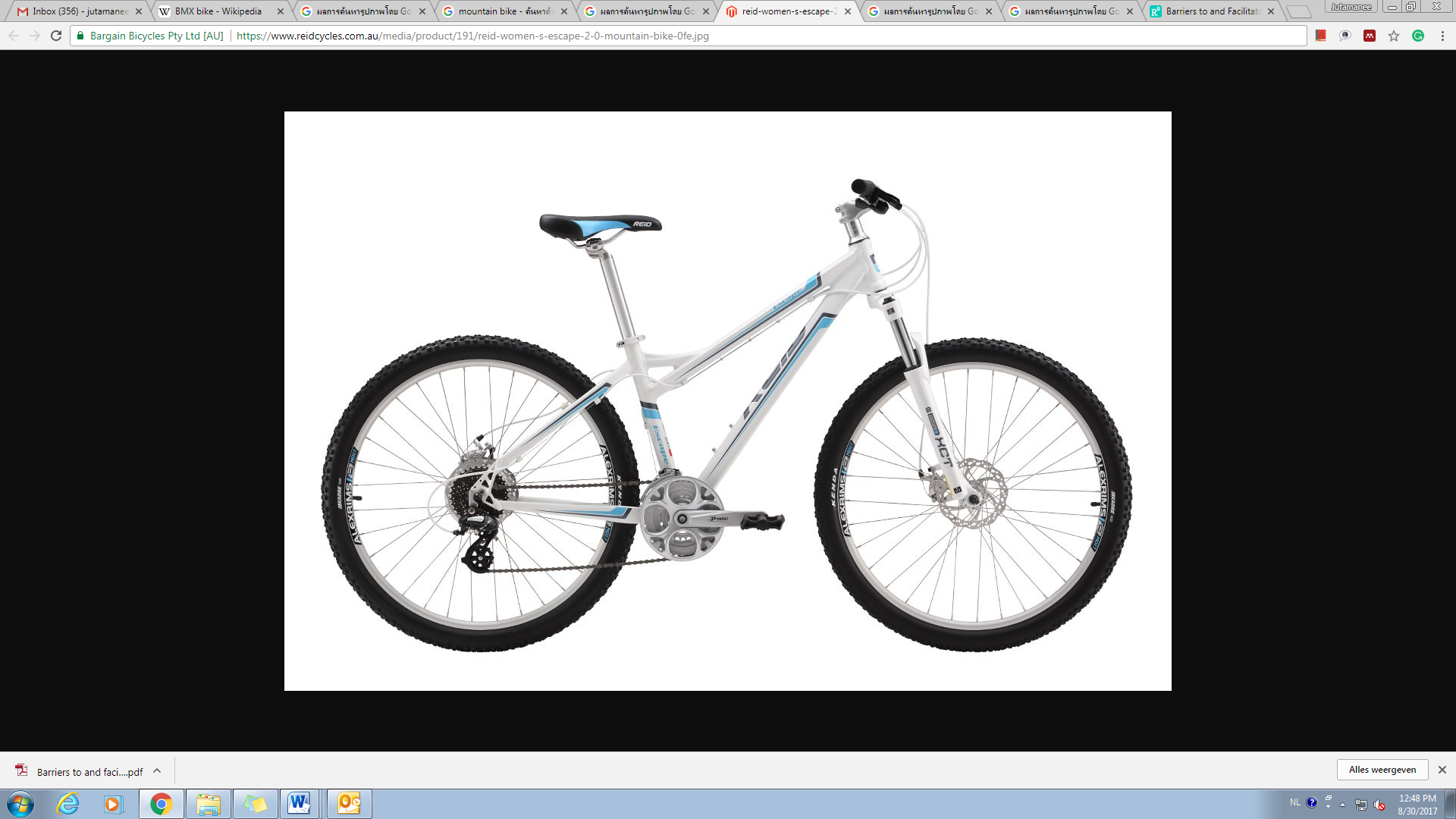Mountain bike | - 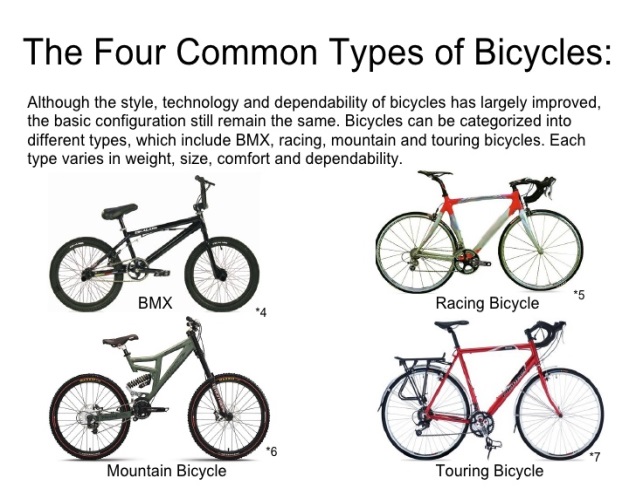Touring bike - 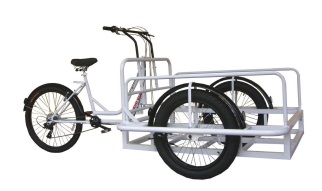   Cargo bike | 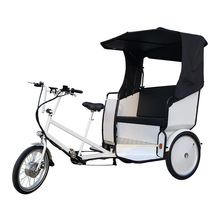   - Rickshaw bike |
| --- | --- | --- | --- |

- Other, namely: ____________________________________________________

_____________________________________________________________________________________________________________________________________

35. When cycling , what kind of shoes do you use?

- Adapted shoes, please describe how it is adapted: _____________________

__________________________________________________________________

__________________________________________________________________

- Use of daily shoes
- Other, namely:___________________________________________________

__________________________________________________________________

36. Did your insurance company compensate the costs of your adapted/cycling prosthesis?

| - No | - Yes |
| --- | --- |

**Part 5: Questions regarding barriers and facilitators for cycling**

37. Have you experienced any barrier when cycling?

| - No. Go to question 39 | - Yes |
| --- | --- |

| \| 38. What is/are the barrier(s) for you to ride a bicycle?(Multiple answers are possible) \| \| \| --- \| --- \| \| I am hindered to ride a bicycle because  of: \| - Lack of energy/effort - Pain at:_______________ - Wound/Injury at:______________ from:______________ - Discomfort while cycling - Poor health conditions - Lack of time - Lack of motivation - Afraid of being injured - Feeling embarrassed about my appearance while cycling - Lack of health improvement from cycling - Feeling too old to cycle - Lack of fun from cycling - Lack of reasons to cycle - Lack of family members who are cycling - Lack of friends who are cycling - Lack of support/encouragement from friends/family/ care taker - Lack of support/encouragement from medical/rehabilitation practitioners - Lack of access to dressing rooms (changing clothes/having a shower) - Lack of rest areas (e.g. Benches) - Potholes in the street - Lack of parking for bicycle - Lack of cycling paths/lanes - Excessive crime in neighborhood or fear of crime in neighborhood - Cars driving too fast on the road - Excessive car traffic in my community - Lack of traffic lights or cross signals for cycling - Lack of adequate street lighting at night - Loose dogs in community - Bad weather (hot, rain) - Pollution - Not owning a bicycle - High cost of cycling equipment - High costs of cycling prosthesis or high costs to adapt prosthesis - High costs of cycling training - Lack of knowledge or skills how to cycle before the amputation - Lack of knowledge or skills how to cycle after the amputation - Lack of information where to cycle - Daily prosthesis problems, prosthesis prevents me from cycling - Bicycle problems, the bicycle is not suitable for conditions - Too close to cycle to destination - Too far to cycle to destination - Other, namely: ______________________________________ \| |
| --- | --- | --- | --- | --- |

| 39. What is/are the facilitator(s) for you to ride a bicycle?(Multiple answers are possible) | |
| --- | --- |
| I ride a bicycle  because of: | - Increasing / maintaining health/physical fitness - Increasing/ maintaining strength - Controlling weight - Having fun/ relaxation - Increasing/maintaining self-confidence - Learning new skills - Increasing/maintaining independence - Accepting disability - Learning how to deal with disability/ assistive device - Increasing/ maintaining social contacts - Support/encouragement from family - Support/encouragement from friends - Support/encouragement from personal care taker - Support/encouragement from medical/rehabilitation practitioners - Support/encouragement from buddies with amputation - Competition/winning - Work - Adequate dressing rooms (changing clothes/ shower) - Adequate rest areas (e.g. benches) - Good quality streets - no potholes - Adequate parking for bicycles - Adequate cycling paths/ lanes - Safe neighborhoods - low crime - Cars driving with appropriate speed on the road/not too fast - Good traffic/not many cars on the road - Adequate traffic lights or cross signals for bicycle - Adequate street lighting at night - No/a few loose dogs in community - Good weather - No pollution - Affordable costs of cycling equipment/accessories - Affordable costs of cycling prosthesis/adapted prosthesis - Affordable costs of cycling/ training program - Free adapted/prosthesis for cycling - Free adaptation of bicycle - Free cycling training - Knowing how to cycle - Knowing where to cycle - Good satisfaction of daily prosthesis - Having a bicycle that fits with my conditions - Appropriate distance to destination/not too far or too close - Other, namely: ________________________________________ |

40. What wishes do you have regarding cycling and prosthesis for people with lower limb amputation?

__________________________________________________________________________________________________________________________________________________
