# Supplementary material for: Cycling of people with a lower limb amputation in Thailand
Source: PLoS One. 2019 Aug 2;14(8):e0220649. doi: 10.1371/journal.pone.0220649 (PMC6677311; doi:10.1371/journal.pone.0220649)
Supplement: S2 Appendix — (DOCX) [file pone.0220649.s002.docx]

# **S2 Appendix 2.Item score of barriers and facilitators.**

|  | Cyclist  (n=197 ) | | Non-cyclists  (n=225) | |
| --- | --- | --- | --- | --- |
|  | **n** | **%** | **n** | **%** |
| Barriers: |  |  |  |  |
| Lack of energy/effort | 9 | 4.6 | 8 | 3.6 |
| Pain | 24 | 12.2 | 3 | 1.3 |
| Wound/Injury | 9 | 4.6 | 3 | 1.3 |
| Discomfort while cycling | 18 | 9.1 | 31 | 13.8 |
| Poor health conditions | 9 | 4.6 | 13 | 5.8 |
| Lack of time | 23 | 11.7 | 19 | 8.4 |
| Lack of motivation | 12 | 6.1 | 20 | 8.9 |
| Afraid of being injured | 12 | 6.1 | 33 | 14.7 |
| Feeling embarrassed about my appearance while cycling | 3 | 1.5 | 4 | 1.8 |
| Lack of health improvement from cycling | 0 | 0.0 | 2 | 0.9 |
| Feeling too old to cycle | 4 | 2.0 | 5 | 2.2 |
| Lack of fun from cycling | 5 | 2.5 | 4 | 1.8 |
| Lack of reasons to cycle | 4 | 2.0 | 23 | 10.2 |
| Lack of family members who are cycling | 2 | 1.0 | 3 | 1.3 |
| Lack of friends who are cycling | 4 | 2.0 | 3 | 1.3 |
| Lack of support/encouragement from friends/family/ care taker | 2 | 1.0 | 5 | 2.2 |
| Lack of support/encouragement from medical/rehabilitation practitioners | 6 | 3.0 | 3 | 1.3 |
| Lack of access to dressing rooms (changing clothes/having a shower) | 2 | 1.0 | 0 | 0.0 |
| Lack of rest areas (e.g. Benches) | 1 | 0.5 | 0 | 0.0 |
| Potholes in the street | 15 | 7.6 | 9 | 4.0 |
| Lack of parking for bicycle | 6 | 3.0 | 1 | 0.4 |
| Lack of cycling paths/lanes | 20 | 10.2 | 7 | 3.1 |
| Excessive crime in neighborhood or fear of crime in neighborhood | 6 | 3.0 | 4 | 1.8 |
| Cars driving too fast on the road | 17 | 8.6 | 9 | 4.0 |
| Excessive car traffic in my community | 19 | 9.6 | 9 | 4.0 |
| Lack of traffic lights or cross signals for cycling | 9 | 4.6 | 4 | 1.8 |
| Lack of adequate street lighting at night | 8 | 4.1 | 4 | 1.8 |
| Loose dogs in community | 13 | 6.6 | 3 | 1.3 |
| Bad weather (hot, rain) | 12 | 6.1 | 2 | 0.9 |
| Pollution | 10 | 5.1 | 4 | 1.8 |
| High cost of cycling equipment | 5 | 2.5 | 5 | 2.2 |
| High costs of cycling prosthesis or high costs to adapt prosthesis | 6 | 3.0 | 1 | 0.4 |
| High costs of cycling training | 0 | 0.0 | 1 | 0.4 |
| Lack of knowledge or skills how to cycle before the amputation | 0 | 0.0 | 16 | 7.1 |
| Lack of knowledge or skills how to cycle after the amputation | 2 | 1.0 | 10 | 4.4 |
| Lack of information where to cycle | 2 | 1.0 | 2 | 0.9 |
| Not owning a bicycle | 8 | 4.1 | 23 | 10.2 |
| Daily prosthesis problems, prosthesis prevents me from cycling | 9 | 4.6 | 20 | 8.9 |
| Bicycle problems, the bicycle is not suitable for conditions | 7 | 3.6 | 8 | 3.6 |
| Too close to cycle to destination | 4 | 2.0 | 0 | 0.0 |
| Too far to cycle to destination | 10 | 5.1 | 7 | 3.1 |
| Other barriers* | 22 | 11.2 | 32 | 14.2 |
| Facilitators: |  |  |  |  |
| Increasing / maintaining health/physical fitness | 132 | 67.0 | 79 | 35.1 |
| Increasing/ maintaining strength | 123 | 62.4 | 79 | 35.1 |
| Controlling weight | 68 | 34.5 | 36 | 16.0 |
| Having fun/ relaxation | 90 | 45.7 | 36 | 16.0 |
| Increasing/maintaining self-confidence | 41 | 20.8 | 18 | 8.0 |
| Learning new skills | 32 | 16.2 | 15 | 6.7 |
| Increasing/maintaining independence | 52 | 26.4 | 27 | 12.0 |
| Accepting disability | 47 | 23.9 | 24 | 10.7 |
| Learning how to deal with disability/ assistive device | 31 | 15.7 | 14 | 6.2 |
| Increasing/ maintaining social contacts | 45 | 22.8 | 16 | 7.1 |
| Support/encouragement from family | 22 | 11.2 | 23 | 10.2 |
| Support/encouragement from friends | 17 | 8.6 | 18 | 8.0 |
| Support/encouragement from personal care taker | 9 | 4.6 | 16 | 7.1 |
| Support/encouragement from medical/rehabilitation practitioners | 9 | 4.6 | 16 | 7.1 |
| Support/encouragement from buddies with amputation | 9 | 4.6 | 14 | 6.2 |
| Competition/winning | 3 | 1.5 | 5 | 2.2 |
| Work | 13 | 6.6 | 5 | 2.2 |
| Adequate dressing rooms (changing clothes/ shower) | 3 | 1.5 | 2 | 0.9 |
| Adequate rest areas (e.g. benches) | 12 | 6.1 | 7 | 3.1 |
| Good quality streets - no potholes | 29 | 14.7 | 14 | 6.2 |
| Adequate parking for bicycles | 27 | 13.7 | 12 | 5.3 |
| Adequate cycling paths/ lanes | 29 | 14.7 | 19 | 8.4 |
| Safe neighborhoods - low crime | 36 | 18.3 | 17 | 7.6 |
| Cars driving with appropriate speed on the road/not too fast | 39 | 19.8 | 19 | 8.4 |
| Good traffic/not many cars on the road | 35 | 17.8 | 24 | 10.7 |
| Adequate traffic lights or cross signals for bicycle | 18 | 9.1 | 16 | 7.1 |
| Adequate street lighting at night | 23 | 11.7 | 11 | 4.9 |
| No/a few loose dogs in community | 37 | 18.8 | 11 | 4.9 |
| Good weather | 56 | 28.4 | 26 | 11.6 |
| No pollution | 44 | 22.3 | 14 | 6.2 |
| Affordable costs of cycling equipment/accessories | 19 | 9.6 | 15 | 6.7 |
| Affordable costs of cycling prosthesis/adapted prosthesis | 7 | 3.6 | 20 | 8.9 |
| Affordable costs of cycling/ training program | 4 | 2.0 | 12 | 5.3 |
| Free adapted/prosthesis for cycling | 12 | 6.1 | 24 | 10.7 |
| Free adaptation of bicycle | 6 | 3.0 | 22 | 9.8 |
| Free cycling training | 4 | 2.0 | 20 | 8.9 |
| Knowing how to cycle | 38 | 19.3 | 30 | 13.3 |
| Knowing where to cycle | 31 | 15.7 | 9 | 4.0 |
| Good satisfaction of daily prosthesis | 49 | 24.9 | 19 | 8.4 |
| Having a bicycle that fits with my conditions | 47 | 23.9 | 25 | 11.1 |
| Appropriate distance to destination/not too far or too close | 57 | 28.9 | 27 | 12.0 |
| Other facilitators | 5 | 2.5 | 6 | 2.7 |

***** Others barriers were: Cannot flex the knee fully or at the same peace as the sound side (n=13), do not have prostheses (n=8), prosthetic foot slipping off the pedal (n=9), have health conditions, so cannot ride the bike (n=8)such as stroke, bilateral LLA, hip and knee problems, afraid/experienced of falling or problems with balance (n=6), socket trim-line caused skin abrasion while cycling (n=2), sweating while cycling and needed to take off the prosthesis (n=1), stump slipped out of the socket while cycling (n=3), prosthesis cannot be used for cycling (n=1), bicycle is not suitable (n=1), cuff suspension became too tight when cycle (n=1).
